# Meat Traceability: Traditional Market Shoppers’ Preferences and Willingness-to-Pay for Additional Information in Taiwan

**DOI:** 10.3390/foods10081819

**Published:** 2021-08-06

**Authors:** Ardiansyah Azhary Suhandoko, Dennis Chia-Bin Chen, Shang-Ho Yang

**Affiliations:** 1 International Master Program of Agriculture, National Chung Hsing University, No. 145 Xingda Rd., South District, Taichung 40227, Taiwan; ianazhary@smail.nchu.edu.tw; 2 Massey College of Business, Belmont University, 1900 Belmont Boulevard, Nashville, TN 37212, USA; dennis.chen@belmont.edu; 3 Graduate Institute of Bio-Industry Management, National Chung Hsing University, No. 145 Xingda Rd., South District, Taichung 40227, Taiwan

**Keywords:** traditional market, traceability, willingness-to-pay, pork, Taiwan

## Abstract

Due to food scandals that shocked the retailer markets, traceability systems were advocated to regain consumers’ confidence and trust. However, while traceability systems can be more easily explored in modern markets, almost no traceability system can be found in traditional markets in Taiwan, especially when buying meat products. This study explored the preference and the willingness-to-pay (WTP) for traceability information of pork products in traditional markets in Taiwan. The random utility theory (RUT) with the contingent valuation method (CVM) was adopted to examine the total of 1420 valid responses in Taiwan. Results show that 80% of traditional market consumers are willing to pay more for traceability information of pork products. Specifically, when consumers (1) know the market price of pork, (2) do not often buy food in the traditional market, (3) live in south or north regions of Taiwan, (4) have a flexible buying schedule, (5) are aware of food safety due to frequently accessing health-related content through media, or (6) think pork grading is very important, they would tend to choose meat products with traceability information. The implication of this study suggests that there is an urgent desire for food safety labeling and traceability information system in traditional markets in Taiwan. Especially, those who usually shop in the higher-price markets are willing to pay the most for this traceability information.

## 1. Introduction

In the current industrialized and mass-produced food era, consumers have heightened safety awareness of the food they buy [1,2,3]. This attention often translates to the purchase behavior of safer products [4,5,6]. Furthermore, Taiwanese citizens particularly showed interest in food safety over time [7], especially when buying food in modern markets. These safety concerns stem from prior labeling deceptions, illicit ingredients, and food scandals around the globe. Therefore, the implementation of a food safety concept is necessary [8,9,10,11] since these concerns can significantly affect consumers’ choices [12]. In Taiwan, past scandals, including avian influenza cases (2015) [13], mad cow disease, dioxin defilement, high percentages of pesticides remaining on agriculture products (the 1990s) [14,15], a codling moth larvae outbreak (1990s) [16,17], and the prominence of foot-and-mouth disease (1997) [18,19], lessen citizens’ trust in food safety [20]. Therefore, food-safety-oriented consumers must be guaranteed safety and quality as they become more selective in buying.

When shoppers take food safety into account, they automatically consider food quality. These two dimensions often blend when shopping for food [21,22]. These concerns could be addressed through additional product information, certifications, and standards [23], as they can increase shoppers’ confidence and their willingness-to-pay (WTP) [7,24]. The presence of these certifications parallels the urgency in applying a traceability system [25]. Therefore, the importance of a traceability system is quite high [26], particularly for producers who aspire towards excellence in food safety and food quality [11,27].

Traceability certification encompasses a system that allows entities along the supply chain to trace forward and back information, including the beginning to final production, multiple stages of processing, and multi-levels of distribution, with the inclusion of the dimensions of breadth, depth, and precision [28,29,30,31,32,33,34]. Due to its importance to the food industry, the most advanced traceability systems implement the Internet of Things (IoT) to detect problems from farm to table through the utilization of wearable devices (e.g., mobiles) in real-time [35]. From the consumers’ perspective, traceability positively impacts the consumers’ confidence level as observed in prior research [10,30,31,36,37,38,39,40]. Moreover, traceability systems saw years of development worldwide since 2002 for the US [31,41], since 2003 for Canada [42], and since 2005 for the EU [43,44,45,46]. In Taiwan, traceability has been discussed since 1990 with the Taiwan Good Agricultural Practice (TGAP) and the Good Manufacture Practice (GMP) [47]. However, Taiwan Agriculture and Food Traceability System (TAFTS) was officially established in 2004 [14,47,48]. The history of these systems demonstrates how traceability is favored in this modern era. Considering all these food safety concerns and development, Taiwanese consumers have critical perceptions on this matter, and those perceptions affect their WTP [7,20,49] when purchasing a product in certain markets. These traceability’s positive impacts on consumers are found in restaurants, [25,33] modern markets [43,50], and supermarkets [10,49]. As to this day, its impact of traceability on consumers’ preference in traditional markets is still limited.

The exigency of further traceability research in traditional markets is situated on the markets’ importance to the Taiwanese economy (USD 5–6 billion annually in 2020 and increasing) [51,52]. An estimated 50,000 stalls are employing approximately 100,000 laborers [53]. Moreover, traditional markets have special characteristics attracting the general buyer more than other markets. These attributes include the chance to haggle the price [54,55,56,57], the ability to build a connection with vendors (increase social scope), the perceived higher quality level [58,59,60,61], and the perceived freshness [62]. Looking at these aspects, meat became the most purchased product in traditional markets, accounting for over 50% of purchases [62]. This might be a result of the cheaper prices, the availability to purchase specific cuts of meat, and the freshly slaughtered selections [55]. Moreover, in the last decade, the Taiwanese increased meat spending in their budget [63]. Especially, pork’s popularity in Taiwan increased, which secures Taiwan as the fifth highest in the world in terms of consumption of pork, with 45 kg per capita each year [64].

Although meat traceability applications show promising results [27,43,65], prior research at the consumer stage was focused on consumers’ attitudes, certainty, and acceptance of traceability [40,47,66,67,68,69]. However, when it comes to consumers’ preferences (WTP), most observations of the positive effect of traceability centered around consumers of multiple other kinds of markets than traditional ones [10,25,33,43,49,50]. Lastly, due to the currently limited traceability research on pork products [50,70,71,72,73,74,75], this study which observes consumers’ preference on traceability impact in Taiwanese traditional market for pork products helps fill the gap in research on this topic.

This research was conducted using a contingent valuation method to measure the consumers’ fondness (or WTP) and how much more they will pay for pork with traceability information in the Taiwan traditional market [76,77]. The research consisted of questions posed in hypothetical market conditions [78,79,80]. The questionnaire has an open-ended format, which lets the respondents reply with their own words in assorted dimensions, and it is commonly used for assessing WTP with an abundant amount of information [81,82,83]. Furthermore, to understand the relationships among consumers’ daily habits and their preferences in the market, this study employed RUT [84,85,86,87] while utilizing CVM to estimate the WTP of a non-marketed item [88]. In short, this study observed the effect of traceability information for pork on traditional market shoppers’ preferences and WTP in Taiwan. Particularly, this research describes the types of consumers sorted by their socio-economic history, shopping habits, and other environmental attributes at both the lower and the higher levels of WTP.

## 2. Materials and Methods

This traditional market research was conducted across Taiwan in 2015. In an attempt to support the more traditional market in sustaining their economic share and in competing with other types of markets (as the growth of modern markets is to keep mounting up [89,90,91]), improving the status quo of traditional markets became one of the foundations of the topic selection. To express consumers’ WTP and their preferences, the CVM (stated preference method) [82,92] was utilized in this hypothetical market situation [88]. The CVM decision allows a monetary valuation to be applied to examine non-marketed items or attributes from a service product [76,88,93,94]. The WTP calculates both the median and the mean from the collected responses of simulated-market questionnaires reflecting the consumer’s budget perception in the real market situation [77,83]. Furthermore, several prior studies stated that the differences are not significant between the real-market WTP valuation and a hypothetical situation [78,79]. A further elaboration of the materials and methods is offered below.

### 2.1. Theoretical Model Used

When consumers buy a product in a traditional market, they usually have many considerations, starting from their daily shopping habits to the atmosphere of traditional markets. The magnitude of these considerations might be small or large based upon the consumers’ utility. This utility can be modeled by adopting the Lancaster consumer theory of RUT [85]. Moreover, this theory can imitate the rational act of consumers going to the market and choosing the option they think most matches their preference (highest utility) [86,87,95]. Since this research applies a non-existent attribute/product (pork belly with traceability information), the application of this theory fits those consumers who pay attention to price and who tend to find utility from an item’s characteristics or attributes rather than the item itself [85,96]. The framework of RUT helped us model every shopper’s utility as a decision-maker in a given hypothetical market situation [84].

The RUT method models the substitute product as a linear function of several attributes. In the research, the independent variables were categorized into three groups ((A) attributes regarding the consumer’s habits in traditional markets, (B) attributes related to pork attributes and their safety, and (C) attributes related to the consumer’s socio and economic profile) and formed the mathematical RUT model (1) below:

*U_ij_* = *β_k_ X_ijk_* + *ε_ij_* (1)

where *U_ij_* represents the utilization of *_i_* consumer for pork belly item *_j_* with the application of traceability information, *β* (utility or preference weights) represents the vectors of coefficients that are homogeneous among traditional market visitors in Taiwan, and *X_ijk_* represents the *_k_* attribute for pork belly item *_j_* of the *_i_* consumer. Finally, the *ε_ij_* (random residue) represents the random component that encompasses the asymmetric factors and the unobserved variance that might impact the genuine utility located throughout consumers [73,97,98], which are independent and identically distributed (*i.i.d.*), and adheres to the type I extreme value distribution and follows the error term differences of logistic distributions [99]. In short, this theory models the decision-maker’s ability to acquire a particular degree of utility from every alternative [66].

### 2.2. Participants and Survey

The data collection was conducted during July and August of 2015. Based upon previous research, pork belly was specified as the meat product in the questionnaire. Moreover, the determination of the prices and the meat product was conducted during the market research phase. The difference of prices and prominent meat products through the island vary, thus this phase assisted the survey in matching the conditions in Taiwanese perception during the research time. Furthermore, freshly slaughtered swine represented more than 50% of the meat bought from traditional market vendors [100], and pork belly was so favored that the government charged a tariff of up to 40% [64].

The survey’s design provided a starting price point. The lower level was 110 TWD per 600 g, the middle level 130 TWD per 600 g, and the upper level 150 TWD per 600 g, as this range was comparable with previous studies’ findings and market conditions from the north to the south of Taiwan [101]. Therefore, this design enabled us to model the value of pork belly with traceability information at these price levels.

While the price was charted into three levels, the questionnaire presented the consumers with two choices initially, which included (a) “do know the price” and (b) “do not know or unsure about the price”. The option for “do know the price” was further subdivided into (i) 110 TWD (~USD 3.94) per 600 g, (ii) 130 TWD (~USD 4.66) per 600 g, and (iii) 150 TWD (~USD 5.38) per 600 g. The option for (b) “do not know or unsure about the price” was assigned the middle level, which was (iv) 130 TWD (approximately USD 4.66) per 600 g. These prices were selected to keep the monetary values consistent with the particular product and markets [102].

When the survey design was completed, the next phase included randomly distributing and sampling in dual channels. The first one was (1) a direct channel, which was conducted at traditional markets and train stations by providing the survey on an electronic tablet (handily utilized without redundant questions and a situation sheet) with a reward administration (a stainless steel cutlery knife) at the end of survey session. The second channel was (2) an indirect channel, where the survey was administered online (through social media, e.g., Facebook) by completing the survey through a webpage. The survey was designed and deployed through the SurveyMonkey platform.

Once the respondents began the survey, they received background information in the form of a scenario to put their state of mind in a shopping situation within a traditional market for pork belly with traceability information. After reading the information, they completed the screening questions which asked (i) “Have you gone to any Taiwan traditional market in the past year?” and (ii) “Have you purchased any fresh meat items at any Taiwan traditional market in the past year?”. Respondents were removed from further analysis whenever they responded “No, I have not gone/have not purchased”, “No, I do not believe”, or “I am unsure”. This phase was critical to mitigate sample bias and to accurately estimate the WTP [103].

The questionnaire involved answering questions surrounding 23 independent variables defined in Table 1.

Further, the dependent variable consisted of both the positive WTP and the WTP of traceability information. In the end, 1420 valid responses were collected. After the data were retrieved, they were cleaned and organized neatly. In summary, the details of all these independent and dependent variables is further spelled out in the sub-section of the theoretical model used and the sample distribution section. The analysis phase is described below.

### 2.3. Data Analysis

The data analysis centers on the WTP probability for traceability information of traditional market consumers. Because the utility of the consumers might be dependent upon distinctive attributes (such as personal habits or environmental conditions), the effect of traceability information on pork belly in Taiwan’s traditional market can be predicted through econometric analysis. The data collected consisted of both continuous and ordinal types, therefore the positive WTP for the observed attribute (pork belly traceability) could be analyzed by the application of the logit model. The notation for its mathematical equation is written on Equation (2) below:

(2) $${p=\mathrm{pr}\;(y}_{i}{=1\;|\;x}_{i})=F\left(x^{\prime }\beta \right)=\frac{e^{x^{\prime }\beta }}{{1+e}^{x^{\prime }\beta }}=\frac{{\exp(x}^{\prime }\beta )}{{1+\exp(x}^{\prime }\beta )}$$

where, in Equation (2), $y_{i}=1$ represents the likelihood of a positive preference or WTP, $x_{i}$ represents independent variables (e.g., (A) market-related habit, (B) safety-and-pork-related information, (C) socio-economy profile). Further, within the logit model, the estimation of marginal effect (M.E.) is shown as ∂*p*/∂*xj* = *F*’(*x’β*) *β_j_*.

The next step in data analysis was finding out the extension of WTP. The probability test by the logit model provided the impact of the attributes with the independent variables and whether they manifested positive or negative impacts. Moreover, if the given attributes showed a positive impact, it was possible to run interval regressions to find out how much New Taiwan dollars (TWD) traditional market consumers will pay for the pork belly with traceability information, because interval regression possesses the ability to forecast the WTP within the limits of the 0 to 1 mathematical term of prediction-likelihood [104]. According to the interval barrier of WTP, the application of interval regression in this research was written as notation in Equations (3) and (4) below:

(3) $$y_{i}^{*}{=x}_{i}^{\prime }{\beta +u}_{i}$$

(4) $$\mathrm{Pr}\;\left[a_{j}{\;<\;y}^{*}\;\leq {\;a}_{j+1}\right]=\mathrm{Pr}\;\left[y^{*}\;\leq {\;a}_{j+1}\right]\;-\;\mathrm{Pr}\;\left[y^{*}\;\leq {\;a}_{j}\right]{=F}^{*}\left(a_{j+1}\right)\;{-\;F}^{*}\left(a_{j}\right)$$

In elaborating Equations (3) and (4), $y_{i}^{*}$ was examined to be found inside the (J+1) exclusive to one another as intervals $\left(-\infty ,a_{1}\right]$, $\left(a_{1},a_{2}\right]$, …, $\left(a_{J},\infty \right)$. Provided the consumers’ response from the questionnaire, $y^{*}$ was located and put in consistent intervals, which were $y^{*}\leq \;0$, 1 ${<\;y}^{*}\leq \;3$, ${4\;<\;y}^{*\;}\leq \;6$, …, and $16\;\leq {\;y}^{*}$. Understanding all these terms and all the 23 independent variables, the empiric specs of the pork belly with traceability information found in Taiwan traditional market are shown as Equation (5):

(5) $$y^{*}{=\beta }_{0}{+\beta }_{1}X_{1}{+\beta }_{2}X_{2}{+\ldots +\beta }_{23}X_{23}+\;\varepsilon$$

In elaborating Equation (5), the WTP for traceability information on pork belly is defined as $y^{*}$ and is affected by 23 independent variables, which are defined as $X_{s}$. Other terms are $\beta_{S}$, which is defined for the estimation parameters, and *ε*, which is defined for the unobserved variance.

## 3. Results

The results of this study are discussed in three sub-sections: (1) sample profile, (2) the probability of positive WTP, and (3) the WTP for traceability information. In general, out of the 1420 respondents, of those people who knew the price, 467 people selected (i) 110 TWD (~USD 3.94) per 600 g, 223 people selected (ii) 130 TWD (~USD 4.66) per 600 g, 102 selected (iii) 150 TWD (~USD 5.38) per 600 g. Further, 628 people selected “do not know or unsure about the price” which was translated to (iv) 130 TWD (~USD 4.66) per 600 g. The further results are pointed out in the tables and the paragraphs below.

### 3.1. Sample Profile

Based on Table 2, more than three-quarters of the respondents would spend extra money for traceability information, as their positive WTP was positive TWD. These respondents also showed their WTP reached 7 TWD (~USD 0.25) whenever facing the pork belly with traceability information in Taiwan’s traditional market. Almost 50% considered themselves as a daily cook at home. Most of them considered themselves to be the major buyer’s representative and go to market before 11:00., spending only 30–60 min. Moreover, around 70% of these people believed that traceability qualification is relevant to pork safety. Approximately 40% of them believed that pork grading is necessary to be provided by the sellers and that the fat-lean proportion can increase buyer’s WTP; they also often access mass media to find health-related content. Lastly, from the socio-economy profile, the results showed >50% of the respondents were female, with fewer than 20% of them housewives. The average age was 41 years old with at least 15 years of formal education. Most of them lived in an urban district or around northern Taiwan, had a monthly salary of around 65,000 TWD (~USD 2330) every month, and had at least four people living in the house.

### 3.2. The Outcome of Positive WTP for Traceable Pork

The logit model result of positive WTP for traceable pork is presented in Table 3. According to Table 3, the log-likelihood and the Wald X^2^ values were estimated and valued as −614.55 and 47.82, respectively. This revealed that the overall model specification was valid and significant. Each variable was explained if both coefficients and marginal effect (M.E.) reached a significant level. From the market-related habit, it mattered at what point-of-time consumers shop in a day. Especially, respondents who went to the market in the morning (prior to 11:00) and noon-to-afternoon (11:00–17:00) were significantly different from those who usually shopped during the evening period (after 17:00). These results make sense because traditional market consumers who usually shop after 17:00 are often hurrying to go back home to prepare dinner for the family. Therefore, the traceable pork information may not be as attractive compared to those who shop prior to 17:00. From the safety-and-pork-related side, those who thought a safety certificate is relevant to pork safety were more likely to give positive WTP compared to those who thought it irrelevant. Moreover, respondents who thought that pork grading information is fair to them showed positive WTP for traceability information compared to those who just thought that the pork-grading is either unnecessary or very unnecessary. Lastly, from the socio-economic side, the only variable that showed its significance was age. However, the result showed that the younger Taiwan-traditional-market buyers were more likely to give a positive WTP for traceability information compared to those who were older. In short, the following independent variables which had statistical significance are shown in Table 3: shopping time, safety qualification, pork grading information, and age of the respondents.

### 3.3. The Estimated WTP of Traceability Information

The WTP elicitation for traceable pork could be evaluated through an interval regression model as shown in Table 4. Because of the effect of price knowledge when eliciting WTP, it was important to distinguish whether respondents knew about the current market price for pork in traditional markets. There are four different interval regression examinations shown in Table 4. The results of log-likelihood and Wald X^2^ presented a strong fit by each model examination in each category. Further, most of the consumers in the survey thought that they had knowledge of pork prices in traditional markets (*n* = 792), while others who did not have knowledge about the pork price were slightly lower (*n* = 628).

The elicitation of WTP in the interval regression model could be explained as the actual dollar that respondents want to pay extra for the traceable pork. Digging deeper into the results based on respondents’ selection of price, the first price outlined showed the people who knew the price and chose 110 TWD (~USD 3.94) per 600 g. It potentially demonstrates that there are still a fair number of consumers who face lower pricing in traditional markets in Taiwan. These Taiwanese with a higher monthly income wanted to pay around 2 cents TWD per 600 g (<USD 0.0007) for traceable pork in traditional markets if compared to those who had a lower monthly income. However, the consumer with lower education also desired to pay more, around 36 cents TWD per 600 g (~USD 0.01), if compared to those who had a higher education level. Lastly, whenever respondents thought that pork grading is very necessary to be provided by a pork-butcher, they wanted to pay a premium extra of about 3.5 TWD per 600 g (~USD 0.13) for traceability information if compared to those who thought the pork grading is unnecessary or very unnecessary.

The middle-price selectors showed a significant difference in WTP on many independent variables. The people who knew the market price at 130 TWD (~USD 4.66) per 600 g cared a great deal about the family, as they showed the premium of WTP at about 1 TWD per 600 g (~USD 0.03) whenever they had more members in the household and resided in an urban district. The Taiwanese with a lower monthly income wanted to pay around 3 cents TWD per 600 g (<USD 0.001) for traceable pork in traditional markets if compared to those who had a higher monthly income. Respondents who typically were not housewives and the prevalence of cooking at home contributed to increased WTP of 2.1 TWD per 600 g (~USD 0.07) if they cooked at home more often. This was related to the next increase in WTP by people who always go traditional-market shopping in the noon–afternoon, and they added 2.76 TWD per 600 g (~USD 0.10) more. To end, these respondents seemed not to be housewives, since these respondents added more WTP (~1.87 to 4.50 TWD per 600 g) for pork grading either necessarily or very necessarily provided by the butcher in traditional markets.

There were still a large number of respondents who did not know the pork belly price in traditional markets, thus respondents were provided the average market price at 130 TWD (~USD 4.66) as the starting point of WTP elicitation. These people perceived that they would be likely to pay around 1.24 TWD per 600 g (~USD 0.04) more for traceable pork if the respondents were female. Further, younger respondents were likely to pay about 6 cents TWD more per 600 g (~USD 0.002) for traceability information compared to those who were older. If respondents often received health-related content from mass-media, they would have WTP of about 1.68 TWD per 600 g (~USD 0.06) for traceable pork if compared to those who rarely received health-related information. Moreover, the pork grading information had the biggest impact when respondents did not know about the market price. Respondents who thought pork grading information is very necessary would be likely to pay about 3.02 TWD more per 600 g (~USD 0.11) for traceability information compared to those who thought pork grading information is unnecessary or very unnecessary.

Ultimately, the final section of the results elaborated on Taiwanese who knew and chose a price of 150 TWD (~USD 5.38) per 600 g. These results potentially showed that there were still small groups of consumers who faced higher pricing in traditional markets in Taiwan. These respondents likely had the highest range of WTP rise, which started from 4 to 9 TWD per 600 g (~USD 0.14–0.32). However, consumers who were interested in the fat-lean-proportion information may not have been willing to give a positive WTP for traceability information. This may imply that those who were not interested in fat-lean proportion information would be willing to pay for the traceability information. The inclusion of 6 TWD per 600 g (~USD 0.22) to these respondents’ WTP was added whenever they were male or considered sometimes-major-shoppers. Moreover, if the respondents in this group were not living in central Taiwan and often listened to health content on media, they had an increase of WTP at around 7 TWD per 600 g (~USD 0.25). Then, the highest supplement to their WTP was found if they thought that pork grading is very necessary to be provided by butchers and had the habit of going to the market prior to 11:00 and 11:00–17:00, as they would pay 6–9 TWD more per 600 g (~USD 0.22–0.32). Since the WTP was estimated in each variable, this study further provides a summation of WTP for each category.

### 3.4. The Estimated WTP for Each Category

The significant premium of WTP in this section can be explained as the summation of the significant parameters multiplied by the respective sample mean. For example, if the recurrence of cook-at-home was seven times a week on average, then it was multiplied with the estimated parameter of 0.13 for the WTP of 130 TWD (~USD 4.66) per 600 g. Thus, we obtained a calculated WTP of about 0.91 TWD for recurrence of cook-at-home. Repeating the same step, the summation of premium WTP is shown in Table 5.

Respondents who knew the market price at 130 TWD (~USD 4.66) were more influenced by “recurrence of cook-at-home”, “time-point of shopping (11:00–17:00)”, “necessary and very necessary information on pork grading (provided by butcher as potential service)”, “non-housewife”, “family monthly earnings”, “family size”, and urban district”, and the summation of WTP was about 10.68 TWD (~USD 0.37). However, we saw several factors affecting WTP estimation when respondents did not know about market price in traditional market. Only the factors of “very necessary information on pork grading (provided by butcher as potential service)”, “health-related content from mass-media (often and always)”, “female”, and “age” influenced the WTP elicitation, and the summation of WTP was about 3.48 TWD (~USD 0.12). Especially, note that respondents who knew the market price possibly had WTP for traceable pork at 10.68 TWD, which was about 7 TWD higher than those who did not know the market price in traditional markets.

Respondents who knew the market price at 110 TWD (~USD 3.94) were influenced more by “very necessary information on pork grading (provided by butcher as potential service)”, “educational level”, and “family monthly-earnings”, and the summation of WTP was about 13.69 TWD (~USD 0.48). However, the respondent who experienced and knew the market price at 150 TWD (~USD 5.38) was more of influenced by “major purchaser status (sometimes)”, “time-point of shopping (prior to 11:00)”, “time-point of shopping (11:00–17:00)”, “very necessary information on pork grading (provided by butcher as potential service)”, “fat-lean proportion information (can increase purchase intent)”, “health-related content from mass-media (often and always)”, “male”, and “non-central Taiwan”, and the summation of WTP was about 20.44 TWD (~USD 0.72). If we compared the difference of WTP summation between the categories of the highest and the lowest market price, it also showed about 7 TWD difference.

**Figure 1:**
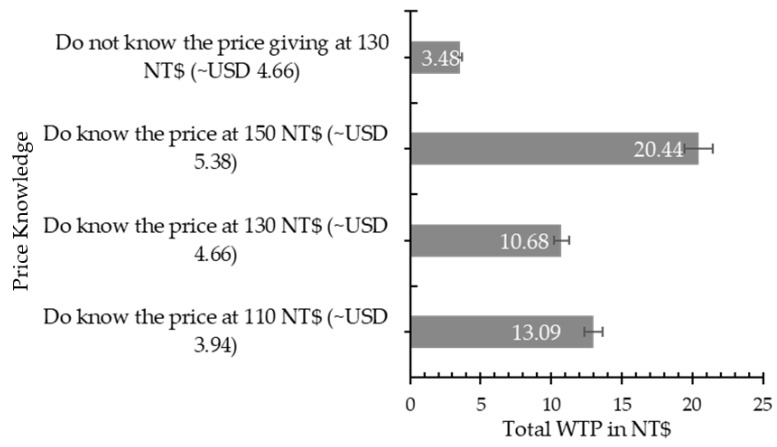
The Total of WTP extra for traceability information (*n* = 1420). Note: The error bars denote 5% of statistical significance.

This final section interprets Figure 1 and shows the significant extra WTP in total. Figure 1 illustrates the total WTP when all attributes were summed based on the price category in traditional markets they visited. This figure shows that every group was significantly different from each other. Furthermore, the largest result of significant WTP extra was from traditional markets that had the highest price from butchers, which represented almost 21 TWD (~USD 0.74). The second largest WTP extra was from traditional markets that had the lowest price from butchers, which revealed about 13 TWD (~USD 0.47). However, note that consumers who knew or did not know about the market price for pork may have had a significant difference in their WTP. Especially, when they did not know about the market price, their WTP for traceable pork was only about 4 TWD (~USD 0.14) for WTP extra. However, if they knew about the market price at 130 TWD (~USD 4.66), their WTP for traceable pork could reach up to 11 TWD (~USD 0.39) for WTP extra.

## 4. Discussion

This section broadly discusses the results of this study that explored the effect of traceability information on meat products in traditional markets in Taiwan. The first part discusses the sample profile. Based on the reported habits, the respondents in this study are active in cooking based on their frequency of cooking at home. The interpretation could be that respondents cook on average once a day. Alternatively, it can also mean that they cook two or three times a day, then skip one day and cook again the next day. This habit leads to their dependability in shopping in the market since most of them are identified as the major shoppers who are responsible for buying the ingredients. They are also mostly time-efficient buyers who prefer buying products in the morning, seeking certain market benefits such as freshness. Regarding safety-and-pork-related information, most of the respondents in the study seem to care about the safety of food. About 40% of the respondents have an interest in pork grading and fat-lean-proportion, which are both related to safety and pork quality. These results are consistent with a prior study showing 60% of respondents put interest in safety guarantee and meat class for animal-derived items [43]. Further, the food safety comprehension seems to come from the mass media, as fewer than half of respondents access health-related materials. Finally, the last part of the profile is the socio-economy profile. Based on previous research, this study profile has a similar pattern with research done in the market either in the modern or the traditional markets. These profiles are written with a monthly salary around 65,000 TWD per month, four members in the household, the average age of 41, with at least 12 years of education, and most self-identify as housewives [47,105,106].

The next discussion concentrates on the likelihood the respondents show positive impacts. Based on the results in Section 3.2, the goodness-of-fit indicators exhibited the model specification used in the study fit the data well. The discussion begins with the market-related habit, which elicits consumers’ positive WTP tendency on the habit related to daytime shopping. This showed that the daytime-preferred respondents give significantly higher positive WTP for traceable pork if compared to the evening shopping schedule. The reason behind this is likely because the evening shoppers usually have work during the daytime and have to rush to quickly buy and return home to prepare dinner for their family [68]. Furthermore, from prior studies, people prefer to buy meat products in the morning for their benefit [107]. From the safety-and-pork-related side, this study finds that people would have positive WTP whenever they were given traceable pork belly in traditional markets. They also think about the safety certification relevance with pork-safety and that pork grading is (moderately or very) necessary and should be included in butcher’s stall. These findings show significantly higher positive impacts than people who do not care about these attributes. They are also consistent with preceding studies which declared that knowledge on meat certification and meat classes connects the food safety perception, as it affects consumers’ decisions towards traceability products [108]. Lastly, from the socio-economy profile, the respondent’s age is discussed. As mentioned before, the results from this study show that WTP is lower as respondents’ ages are older. In other words, the inclusion of traceability information in pork belly has a more positive effect on younger people. The average age was 41 in the study. Therefore, this study found that Taiwanese below age 40 would have a higher WTP when viewing traceability of meat products in traditional markets. This finding is relevant to previous research which mentioned that younger consumers below the age of 35 years old would have more likeliness to buy traceable products compared with other age categories [29]. In short, traceability showcases positive WTP generally towards variables.

The subsequent discussion is centered on the WTP for traceability information. From Section 3.3, the respondents in this research are most likely to know the pork belly price. This finding is likely to be supported by the fact that most of these Taiwanese are identified as major purchasers in the household, making their food almost every day in their homes. Knowing that pork is the most purchased and consumed product in traditional markets [78], as the main shopper, they must know the price of pork because they most likely buy or cook pork daily, weekly, or monthly in their main dish.

The next focus from Section 3.3. is the WTP of respondents who selected 110 TWD (~USD 3.94) per 600 g. These people are considered as common pork buyers because they are the majority of those who know about the market price level. Moreover, their attributes have a significant difference in their WTP. The lower education they have, the more WTP they add. The more income they make, the more WTP they supplement. The WTP is about 3.5 TWD when pork grading has the highest necessity to be provided in the market, and they show the second lowest WTP across all groups of the consumer. The finding on common pork buyers’ income background is compatible with the previous finding, which stated that, when consumers have a preference for traceability and safer products, they increase their WTP, as they have higher income every month [29,105]. However, for the education attribute, the result creates a new finding on traceability preference, whereas the previous finding on education showed the opposite result [105]. The reason behind this could be the general profile of common pork buyers; they are not always people who have an education of more than twelve years. Common pork buyers can be considered just the normal buyers in traditional markets.

The following discussion is centralized on Taiwanese buyers’ WTP who picked the middle price (i.e., 130 TWD (~USD 4.66) per 600 g). These people can be considered career mom pork buyers because their attributes indicate a typical mom who needs to take care of the family and be the family’s backbone. This is supported by the rise in their WTP whenever the family size increases (the larger number they have, the higher WTP they put), when they have purchasing budget management (career woman’s strategy in planning middle price selection, how often to cook at home, how minimal money should be used in the food expenditures), when the location of living differs (more WTP is found in more municipal districts), when they have a shopping schedule concern (because they are a career woman who works the whole day and thus are only able to buy ingredients in the evening), and when they are thinking about the safety and the quality of the food they buy for the family (e.g., they think pork grading is necessary or very necessary). Career mom pork buyers’ preference on evening shopping, number of family members, and pork grading showed (barely cents) an increased WTP. This is supported by a study which stated career woman are found to prefer to buy food in the evening where they can buy food after work hours [109]. Another finding seemed to correspond to prior studies which dictated the more family members there are in the family, the more they care about food quality they serve. In the case of pork grading preferred buyers, they show a higher WTP [110,111]. Although a previous study also mentioned a positive relationship between income and WTP for traceability [24], they were not situated in the price-sensitive class (110 TWD or ~USD 3.94) and still picked middle price (130 TWD or ~USD 4.66). In short, a career woman who takes care of the whole family, including the money flow in purchasing food, stills pick a rational price and still believes in a safe pork product.

The succeeding discussion elaborates upon respondents who did not know the price and who were assigned the middle price. These respondents who did not know the price were younger customers who preferred traceable pork and were curious or showed concern about health-related content from online sources, which affected their pork grading interests. Thus, we labeled these respondents as younger female pork buyers. This curiosity of health and safety materials is relevant with the prevalence of younger consumers’ curiosity and concern levels tending to be elevated. Furthermore, females put more positive impact towards traceable products and meat class than males in the previous research [11,111], which supports these discoveries. 

However, the difference with people choosing the highest price (150 TWD or ~USD 5.38) is the most positive WTP for traceable pork belly for males rather than females. Because of the big range of WTP from 4 to 9 TWD (~USD 0.14–0.32) among their significant attributes and their pick on the premium price, they can be regarded as the health-enthusiast male buyers. This designation is also supported by their higher WTP regarding low occurrence of being the food shopper (which was not often when they were male), buying food in a flexible time (since males are prone to not having particular times to buy food), caring about health content from mass media and pork grading (which increases their WTP), and caring less about fat-lean-proportion. These findings seem novel to the traceability research since previous research identified that it is primarily females who care about health and quality information [11,111]. However, one of the findings on fat-lean-proportion is supported by prior research which showed increased money preference whenever males were less interested in this information to be provided in regard to food [112].

The final part of the discussion focuses on Section 3.4. This part of the discussion concentrates on the highest significant effect of extra WTP. The highest effect was approximately 21 TWD (~USD 0.74) of extra WTP, which might give more possibility of traceable pork belly to be strategized in premium prices of 171 TWD (~USD 5.99) per 600 g, which is highly impacted by the preference on pork grading. This result was also consistent with the previous result, which suggested that, whenever consumers are given food safety certification, their safety awareness leads them to set aside other attributes to pick the premium price ([92,108,113]), especially when the products include traceability information and consumers are scared based on food scandal events in the past [10,47,92,93]. To summarize, whenever Taiwanese consumers acknowledge food safety causation and know the price, they have a much higher WTP for traceable food.

## 5. Conclusions

The safety awareness of Taiwanese keeps rising due to food scandals that happened in the past. This leads to the desire to apply food labels on their food with certifications such as traceability. Traceability might help regain consumers’ confidence and trust while also helping to increase shoppers’ WTP. However, traceability’s positive impact is only currently found in modern markets. As traceability information is not often present in Taiwan’s traditional market, this research provides novel findings.

In general, the application of traceability information on the pork belly product in traditional markets offers a positive effect related to several given attributes among many kinds of market goers. These positive impacts lean towards consumers who are younger buyers, have flexible shopping schedules, are concerned about safety qualification related to pork safety, and perceive pork grading provisions as (commonly) important.

In conclusion, regarding WTP (in TWD), the most favored attribute by all price electors is the attribute of pork grading, since all groups showed the highest increase of WTP. Additionally, whenever consumers have a high recurrence in watching or listening to health-related content in offline or online media, the mindset of food safety and the addition of WTP increase.

The traceable pork belly also pushes increased WTP whenever traditional market visitors prefer to go food shopping in the evening. Another result is that traceability information impacts are sensitive to gender and income gaps. Connecting with the price selection, whenever these respondents acknowledge the price, there is a chance for them to be situated in price options one level higher. Furthermore, the highest market price venue (150 TWD or ~USD 5.38 per 600 g) also has the highest total of extra WTP that enables producers to establish an even higher new price possibility of about 171 TWD (± ~USD 5.99).

Considering these findings, the implication of this research is outlined in two aspects. The first one relates to traditional market vendors. Traditional market vendors may be able to devise their marketing strategy for specific buyers (e.g., ones who choose the premium prices, ones who do not often purchase food in the market, ones who reside in the south or the north area of Taiwan, ones who have a flexible buying schedule, ones who are aware of food safety, ones who frequently access health-related content on online or offline media, and ones who perceive pork grading as very important) and create a new price point of 170 TWD (~USD 5.99) per 600 g. The second direction addresses the government’s role, as these results can be applied to encourage food safety issues along with traceability information and pork grading information via mass media online or offline, especially towards particular buyers based on their sex-gender, shopping schedule, and monthly income gaps.

Finally, the limitation of this study may be the hypothetical perception that the market simulation was utilized for this research. Even with the utilization of CVM or RUT and the screening questions asked, the hypothetical condition might have reached the limit of consumers’ preference, and real market situations may lead to different results. Thus, in future studies, the introduction of “cheap talk” at the beginning of the questionnaire is needed, as it decreases the hypothetical perception [114,115]. The “cheap talk” method can give insight to the respondents before they start the survey by providing them an explanation or script of possible hypothetical bias that could happen, thus they are asked to disregard the conjecture effect and are recommended to situate themselves in a real market situation.

## Figures and Tables

**Table 1:** The attributes regarding each survey question.

Attributes to their habit in traditional markets	The status as a food purchaser within the family	All the time
		Sometimes
		No or never (reference group)
	The duration of traditional market shopping	Less than 30 min (reference group)
		30–60 min
		More than an hour
	The point-in-time of going to traditional markets	Prior to 11:00.
		11:00–17:00.
		In the wake of 17:00. (reference group)
	The recurrence in cooking at the home	
Attributes related to pork and its safety	Safety certificate (relevant with pork safety)	Safety certificate is relevant to pork safety
		Safety certificate has no relevance with pork safety (reference group)
	Pork grading information	Very unnecessary (reference group)
		Unnecessary (reference group)
		Fair
		Necessary
		Very necessary
	Calories and nutrients label	Providing calories and nutrients labels can increase purchase intent
		Providing calories and nutrients labels cannot increase purchase intent (reference group)
	Fat-lean proportion information	Providing fat-lean proportion information can increase purchase intent
		Providing fat-lean proportion information cannot increase purchase intent (reference group)
	The tendency of accessing health-related content from mass media	Accessing health-related content from mass media—never (reference group)
		Accessing health-related content from mass media—seldom (reference group)
		Accessing health-related content from mass media—sometimes (reference group)
		Accessing health-related content from mass media—often
		Accessing health-related content from mass media—almost every time
Attributes to their socio and economic profile	Sex-gender	Female
		Male (reference group)
	Housewife status	Occupancy as housewife status
		Occupancy not as housewife status (reference group)
	Survey completion location	Northern Taiwan
		Central Taiwan
		Southern Taiwan (reference group)
		Eastern Taiwan (reference group)
	Buyers’ origin (rural or urban district)	Urban district
		Rural district (reference group)
	Age	
	Family size	
	Range of family earnings every month	
	Education level	

Source: Assembled by this research.

**Table 2:** Description of variables (*n* = 1420).

Variables		Mean	SD	Type	Measurement
Dependent					
Positive WTP (put extra TWD from 0-up)		83%	0.37	BV	Will put = 1 Otherwise = 0
WTP of traceability information		7	5.69	CV	WTP in TWD
Independent					
Market-related habit					
Recurrence of cook-at-home		7	5.17	CV	Frequency in times
Major purchaser status (All the time)		50%	0.50	BV	All the time = 1 Otherwise = 0
Major purchaser status (Sometimes)		32%	0.47	BV	Sometimes = 1 Otherwise = 0
Time-point of shopping (prior to 11:00.)		43%	0.50	BV	11:00 or before = 1 Otherwise = 0
Time-point of shopping (11:00–17:00.)		22%	0.42	BV	11:00–17:00. = 1 Otherwise = 0
Duration of shopping (30–60 min)		50%	0.50	BV	30–60 min = 1 Otherwise = 0
Duration of shopping (more than an hour)		14%	0.34	BV	≥ 1 h = 1 Otherwise = 0
Safety-and-pork-related information					
Safety qualification (relevant with pork safety)		73%	0.44	BV	Relevant = 1 Otherwise = 0
Pork grading (provided by butcher as potential service)	Fair	30%	0.46	BV	Fair = 1 Otherwise = 0
	Necessary	47%	0.50	BV	Necessary = 1 Otherwise = 0
	Very necessary	15%	0.36	BV	Very necessary = 1 Otherwise = 0
Fat-lean proportion information (can increase purchase intent)		36%	0.48	BV	Increase = 1 Otherwise = 0
Calories and nutrients label (can increase purchase intent)		20%	0.40	BV	Increase = 1 Otherwise = 0
Health-related content from mass-media (often and always)		40%	0.49	BV	Often and always = 1 Otherwise = 0
Socio-economy profile					
Sex-gender		66%	0.47	BV	Female = 1 Otherwise = 0
Housewife status		13%	0.33	BV	Housewife = 1 Otherwise = 0
Age		41	10.49	CV	Age in years
Education level		15	2.31	CV	Education in years
Family monthly-earnings		65	31.42	CV	Salary in 1000 TWD
Family size		4	1.51	CV	Amount of people
Urban district		64%	0.48	BV	Urban = 1 Otherwise = 0
Northern Taiwan		49%	0.50	BV	Northern = 1 Otherwise = 0
Central Taiwan		28%	0.45	BV	Central = 1 Otherwise = 0

Source: Assembled by this research. Note: BV = binary variables, CV = continuous variables, SD = standard deviations.

**Table 3:** Probability of positive WTP for traceable pork (*n* = 1420).

Independent Variables		Dependent Variables	
		Positive WTP	
		*M.E.*	*Coef.*
Market-related habit			
Recurrence of cook-at-home		0.00	−0.01
Major-purchaser status (All the time)		−0.05	−0.34
Major-purchaser status (Sometimes)		0.00	−0.01
Time-point of shopping (prior to 11:00)		0.07 ***	0.54 ***
Time-point of shopping (11:00–17:00)		0.07 ***	0.53 ***
Duration of shopping (30–60 min)		0.00	−0.02
Duration of shopping (more than an hour)		−0.02	−0.16
Safety-and-pork-related information			
Safety certificate (relevant with pork safety)		0.05 **	0.37 **
Pork grading (provided by butcher as potential service)	Fair	0.06 **	0.48 *
	Necessary	0.04	0.26
	Very necessary	0.06 *	0.49
Fat-lean proportion information (can increase purchase intent)		0.02	0.12
Calories and nutrients label (can increase purchase intent)		−0.02	−0.13
Health-related content from mass-media (often and always)		0.03	0.20
Socio-economy profile			
Sex-gender		−0.01	−0.05
Housewife status		−0.01	−0.11
Age		−0.00 ***	−0.02 ***
Education chronicle		0.00	0.02
Family monthly-earnings		0.00	0.00
Family sum		0.00	0.03
Urban district		0.00	0.00
Northern Taiwan		0.01	0.11
Central Taiwan		0.04	0.27
Constant		1.16	
Log-Likelihood		−614.55	
Wald X^2^		47.82 ***	
Classification Predication		83.52%	
Goodness-of-fit		1411.47	
Pseudo R^2^		0.04	

Source: Assembled by this research. Note: M.E. = marginal effect, Coef. = coefficient, *** = 1%, ** = 5%, * = 10% of statistical significance.

**Table 4:** The outcome of interval regression for traceability information (*n* = 1420).

Independent Variables		Dependent Variables			
		Do Know the Price			Do Not Know the Price
		WTP 110 TWD (~USD 3.94)	WTP 130 TWD (~USD 4.66)	WTP 150 TWD (~USD 5.38)	WTP 130 TWD (~USD 4.66)
Market-related habit					
Recurrence of cook-at-home		−0.02	0.13 *	−0.28	−0.04
Major purchaser status (All the time)		−0.34	−1.37	3.03	−0.82
Major purchaser status (Sometimes)		0.45	−0.49	6.44 *	0.02
Time-point of shopping (prior to 11:00.)		−1.18	0.58	8.09 ***	0.96
Time-point of shopping (11:00–17:00)		−1.22	2.76 ***	5.59 *	0.84
Duration of shopping (30–60 min)		0.98	0.61	0.73	−0.37
Duration of shopping (more than an hour)		1.08	−0.50	−1.32	−0.67
Safety-and-pork-related information					
Safety certificate (relevant with pork safety)		0.78	0.96	−1.21	0.82
Pork grading (provided by butcher as potential service)	Fair	0.85	1.01	6.20	1.11
	Necessary	1.27	1.87 *	7.69	1.04
	Very necessary	3.50 **	4.50 ***	9.28 *	3.02 ***
Fat-lean proportion information (can increase purchase intent)		1.04	0.56	−3.70 **	−0.21
Calories and nutrients label (can increase purchase intent)		1.42	−0.37	2.58	−0.76
Health-related content from mass-media (often and always)		0.70	0.67	6.77 ***	1.68 ***
Socio-economy profile					
Sex-gender		−0.03	0.41	−5.57 ***	1.24 **
Housewife status		0.95	−1.97 **	1.08	−1.05
Age		−0.06	0.00	−0.19	−0.06 *
Education level		−0.36 **	0.27	−0.39	0.12
Family monthly-earnings		0.02 *	−0.03 ***	0.00	0.01
Family size		0.38	0.79 ***	−0.23	−0.07
Urban district		−0.11	1.40 *	0.39	0.72
Northern Taiwan		0.92	0.14	−5.50	0.43
Central Taiwan		0.47	0.51	−6.46 *	0.50
Constant		13.69 ***	−6.04	7.73	1.84
Observations (n)		467	223	102	628
Log-Likelihood		−979.48	−450.32	−150.64	−1332.98
Wald X^2^		34.73 ***	64.26 ***	41.86 ***	49.07 ***
AIC		2008.97	950.64	351.28	2715.97

Source: Assembled by this research. Note: Coef. = coefficient, M.E. = marginal effect, *** = 1%, ** = 5%, * = 10% of statistical significance.

**Table 5:** The summation of significant premium WTP (*n* = 1420).

Independent Variables		Mean	Dependent Variables			
			Do Know the Price			Do Not Know the Price
			110 TWD (~USD 3.94)	130 TWD (~USD 4.66)	150 TWD (~USD 5.38)	130 TWD (~USD 4.66)
Market-related habit						
Recurrence of cook-at-home		7	Ø	0.91	Ø	Ø
Major-purchaser status (Sometimes)		1	Ø	Ø	6.44	Ø
Time-point of shopping (prior to 11:00)		1	Ø	Ø	8.09	Ø
Time-point of shopping (11:00–17:00)		1	Ø	2.76	5.59	Ø
Safety-and-pork-related information						
Pork grading (provided by butcher as potential service)	Necessary	1	Ø	1.87	Ø	Ø
	Very necessary	1	3.50	4.50	9.28	3.02
Fat-lean proportion information (can increase purchase intent)		1	Ø	Ø	−3.70	Ø
Health-related content from mass-media (often and always)		1	Ø	Ø	6.77	1.68
Socio-economy profile						
Sex-gender		1	Ø	Ø	−5.57	1.24
Housewife status		1	Ø	−1.97	Ø	Ø
Age		41	Ø	Ø	Ø	−2.46
Education level		15	−5.40	Ø	Ø	Ø
Family monthly-earnings		65	1.30	−1.95	Ø	Ø
Family size		4	Ø	3.16	Ø	Ø
Urban district		1	Ø	1.40	Ø	Ø
Central Taiwan		1	Ø	Ø	−6.46	Ø
Constant		1	13.69	Ø	Ø	Ø
The Significant Extra-WTP			13.09	10.68	20.44	3.48

Source: Assembled by this research. Note: Ø = denotes null symbol as the values do not come statistically significant.

## Data Availability

The datasets generated for this study are available on request to the corresponding author.
